# Breeding for Integrated Pest Management (B-IPM): a new concept simultaneously optimising plant resistance and biocontrol

**DOI:** 10.3389/fpls.2025.1659069

**Published:** 2025-09-16

**Authors:** Mudassir Iqbal, Adam Flöhr, Erik Andreasson, Johan A. Stenberg

**Affiliations:** ^1^ Department of Plant Protection Biology, Swedish University of Agricultural Sciences, Lomma, Sweden; ^2^ Department of Biosystems and Technology, Swedish University of Agricultural Sciences, Lomma, Sweden

**Keywords:** *Aureobasidium pullulans*, biological control, *Botrytis cinerea*, Breeding for Integrated Pest Management (B-IPM), *Colletotrichum acutatum*, *Fragaria vesca*, postharvest disease resistance

## Abstract

Plant breeding for disease resistance typically focuses on the traits that target pathogens, although such traits may antagonise beneficial microbes, thus thwarting any opportunities for biocontrol. In this paper, we propose the concept of Breeding for Integrated Pest Management (B-IPM) which requires the simultaneous optimisation of plant traits that confer resistance to pathogens and facilitation of biocontrol agents. We tested the prospects for B-IPM by screening wild strawberry (*Fragaria vesca*) genotypes for resistance to the detrimental pathogens *Botrytis cinerea* (causing grey mould disease) and *Colletotrichum acutatum* (causing anthracnose disease) and facilitation of the beneficial biocontrol agent *Aureobasidium pullulans*. The plant genotypes showed strong variation in their resistance to the two pathogens and their ability to facilitate biocontrol. However, while the resistance of plant genotypes to both pathogens was strongly correlated, there was no correlation between this and facilitating biocontrol, suggesting that resistance and biocontrol facilitation can be independently optimised to prepare plants for pesticide-free farming.

## 1 Introduction

Domesticated crops typically rely on frequent applications of chemical pesticides to resist pathogens and produce good yields (Sharma et al., 2019). These pesticides may, however, pose risks to biodiversity (Cordero-Bueso et al., 2014; Geiger et al., 2010; Muñoz-Leoz et al., 2011; Salis et al., 2023) and raise serious concerns for human health (Asghar et al., 2016). For these reasons, dramatic policy changes around the world call for a green transformation of agriculture through which chemical pesticides would be phased out. Two main alternative crop protection strategies are available: 1) breeding for resistance (Mendes et al., 2018; Wissuwa et al., 2009), and 2) biological control using beneficial microbes to combat the pathogens (Collinge et al., 2022; Stenberg et al., 2021). However, these two options have historically been pursued in separate scientific fields (i.e., *Plant science* and *Microbiology*) and have never been simultaneously optimised to achieve disease control for food crops. This lack of integration has likely been exacerbated by the assumption that plant resistance traits, particularly broad-spectrum resistance, may inadvertently interfere with the establishment of beneficial microbes (Chen et al., 2015; Grau Nersting et al., 2006; Nerva et al., 2022; Park et al., 2023; Pérez-Jaramillo et al., 2016), making simultaneous optimisation difficult. More specifically, it is often suggested that core immune responses — such as pattern-triggered immunity (PTI) and systemic acquired resistance (SAR) do not effectively distinguish between pathogenic and beneficial microbes, potentially limiting the success of biocontrol agents (Pieterse et al., 2014; Zamioudis and Pieterse, 2012; Zhou and Zhang, 2020). This challenge underlines the need for empirical studies to determine whether resistance and microbe facilitation traits are inherently linked or can be decoupled and optimised independently.

We therefore hypothesise that the simultaneous optimisation of plant resistance and biocontrol should be both possible and feasible. We base this hypothesis on eco-evolutionary theory which suggests that the evolution of wild plant traits results from simultaneous natural selection from among many biotic selectors including both pathogens and beneficial microbes (Angulo et al., 2022). The direction and outcome of such selection may vary in space and time, leading to dynamic geographic mosaics of (co)-evolution (Burdon and Thrall, 2014; Thompson, 2005). Such mosaics uphold trait diversity and increase opportunities to find useful trait combinations, including combinations that simultaneously antagonise pathogens and facilitate beneficial organisms. If this is the case, simultaneous optimisation of broad resistance traits and biocontrol should be possible if the compatible plant traits were to be identified and integrated at an early stage of breeding.

We propose the concept of *Breeding for Integrated Pest Management* (B-IPM) which aims to simultaneously optimise the traits which enable plants to resist pathogens and those that facilitate biocontrol agents. By integrating these objectives, B-IPM aims to utilise the potential of beneficial microbes while maximising plant resistance. This approach could significantly reduce reliance on pesticides, contributing to more sustainable and environmentally-friendly farming practices. We tested the feasibility of B-IPM using wild strawberry genotypes (*Fragaria vesca* L., Rosaceae) and their main pathogens, *Botrytis cinerea* (a necrotrophic fungus that causes grey mould) and *Colletotrichum acutatum* (a hemibiotrophic fungus that causes anthracnose disease). These pathogens are important globally, inflicting major losses on the yields of many fruit crops, including citrus (Daoud et al., 2019), wine grapes (Cosseboom and Hu, 2022; Jiang et al., 2023), strawberries (Petrasch et al., 2019; Wang et al., 2019), and apples (Ma et al., 2018; Moreira et al., 2019). In addition, *B. cinerea* inflicts major losses on various vegetable and grain crops and conifer seedlings in forest nurseries (Kim et al., 2016; Liu et al., 2016; Nielsen et al., 2024). Plants’ mechanisms for resisting these pathogens are complex, relying on quantitative resistance traits (Petrasch et al., 2022).

Wild strawberry has a small genome and is known to show high genetic variation in many agronomy-relevant traits (Shulaev et al., 2011). It has emerged as an increasingly important model species in plant science and a valuable source of wild genetic resources for breeding of Rosaceae crops (Alsheikh et al., 2002; Oosumi et al., 2010; Ruiz-Rojas et al., 2010; Shulaev et al., 2011). Despite some technical limitations such as incomplete genome annotation and a relatively complex gene repertoire — *F. vesca* was selected for this study because it provides a practical and ecologically relevant system for screening resistance and biocontrol traits. It also serves as a valuable bridge between fundamental plant biology and applied breeding within the Rosaceae family. Building on these strengths, our study focused on evaluating resistance to the two pathogens *B. cinerea* and *C. acutatum* in a range of wild strawberry genotypes. At the same time, we evaluated these genotypes’ ability to support the beneficial yeast-like biocontrol agent *Aureobasidium pullulans* (De Bary) G. Armaud, which is known for its antagonistic ability and biological control of various plant pathogens (Di Francesco et al., 2020; Di Francesco and Mari, 2014; Iqbal et al., 2022; Zhang et al., 2010).

Our findings reveal substantial variation between strawberry genotypes in terms of resistance to both pathogens and their ability to support the biocontrol agent. While resistance to *B. cinerea* and *C. acutatum* was highly correlated, no correlation was observed between resistance and facilitation of biocontrol. This suggests that it is possible to independently optimise these traits and thus create elite cultivars that can both defend themselves directly and facilitate microbial “bodyguards.”

## 2 Materials and methods

### 2.1 Plant material

The woodland strawberry is an herbaceous perennial plant found across the Holarctic region. For the purpose of this study, we selected 16 from a collection of 86 distinct genotypes sourced from wild populations in Uppsala County, Sweden and collected in the spring of 2012 (Egan et al., 2018; Muola et al., 2017; Weber et al., 2020a, Weber et al., 2020b) (**Table 1**). Selection was based on results from previous screenings for resistance against pest insects, ensuring representation of a broad range of resistance levels (Weber et al., 2020a, Weber et al., 2020b). The plant collection was maintained in an outdoor common garden at the Swedish University of Agricultural Sciences (SLU) Campus Alnarp (geographic coordinates: 55.66040521759027, 13.084215568092686) until propagation of experimental plants for this study began. Plastic pots were filled with 500 g of potting soil (Emmaljunga, Vittsjö, Sweden) and clonal propagation was conducted over several generations to achieve approximately 80 runners per plant genotype to be used in this experiment. These runners were then used to propagate new experimental plants which were cultivated in a greenhouse under conditions of 22 ± 2°C temperature and 70 ± 5% relative humidity until the experimental setup described below was established (paragraph: *Experimental design*).

**Table 1:** Coordinates (World Geodetic System) of the 16 woodland strawberry (*Fragaria vesca*) genotypes used in the study.

Plant genotype ID	Longitude	Latitude
01A	17.4607833	60.58625
04A	17.4682	60.4927166
05A	17.6124333	60.4346833
06A	17.7712667	60.4465167
08F	18.5696667	60.3076166
10A	17.5596667	60.3738
12F	18.0151833	60.304
18A	18.0061166	60.1617667
19A	18.2141	60.1582667
19F	18.1865667	60.1976166
20F	18.3433333	60.1018
23A	17.4720834	60.1559666
34F	18.2901	59.88625
40A	18.0445	59.7585834
43A	17.34075	59.8165667
48F	17.3085166	59.6393334

### 2.2 Fungal strains, maintenance, and formulation preparation

The *A. pullulans* strain AP-SLU6 originally isolated from woodland strawberry (Iqbal et al., 2021), the *B. cinerea* strain B05.10, and *C. acutatum* were cultured and maintained on potato dextrose agar (PDA) medium (Oxoid; Basingstoke, Hampshire, England) at 25°C under dark conditions. These fungal strains were revived from stock cultures preserved in 20% (wt/vol) glycerol at -80°C.

The *A. pullulans*, *B. cinerea*, and *C. acutatum* cultures were incubated on PDA Petri dishes at 25 °C for a duration of two weeks under dark conditions. Conidia produced by these fungi were harvested by adding 7–10 ml of sterile water to the fungal culture, followed by gentle scraping of the mycelium surface using a spreader (Iqbal et al., 2021; 2023a). Conidia concentrations were then measured and adjusted to 10^7^ CFU/mL for *A. pullulans*, 2 × 10^5^ for *B. cinerea*, and 10^6^ CFU/mL for *C. acutatum*. These measurements were made using a hemocytometer (Hausser Scientific, Horsham, PA) under a light microscope (Laborlux12, Leitz, Germany).

### 2.3 Experimental design

To test for plant genotypic variation in resistance to the pathogens *B. cinerea* and *C. acutatum* and investigate whether the plant genotypes varied in their ability to facilitate microbial biocontrol using *A. pullulans*, we conducted a large full-factorial experiment in a greenhouse (22°C; 70% RH). The experiment was conducted using a complete block design and lasted for 12 weeks. Eight treatments, representing different biocontrol and control treatments (**Table 2**) were applied to each of the 16 *F. vesca* genotypes. For every treatment–genotype combination, 10 biological replicates were included. This resulted in a total of 16 genotypes × 8 treatments × 10 replicates = 1280 individually treated potted plants, with each plant serving as an independent data point in the analysis. Pre-application treatments were included to allow sufficient time for colonization of plant tissues or potential induction of plant responses before the subsequent application of another organism. Accordingly, pre-applications of *A. pullulans*, *B. cinerea*, or *C. acutatum* were conducted 48 hours before the respective second application. This setup was designed to simulate both preventive (pre-treatment) and curative (post-treatment) scenarios, allowing us to assess differences in biocontrol efficacy and plant-pathogen interactions under contrasting temporal dynamics.

**Table 2:** Details of the eight (T1-T8) experimental treatment types and combinations applied to strawberry plants in the study.

Treatment	Pre-application			Post-application		
	*Ap*	*Bc*	*Ca*	*Ap*	*Bc*	*Ca*
T1	+	–	–	–	–	–
T2	+	–	–	–	+	–
T3	+	–	–	–	–	+
T4	–	+	–	–	–	–
T5	–	+	–	+	–	–
T6	–	–	+	–	–	–
T7	–	–	+	+	–	–
T8	–	–	–	–	–	–

Pre- or post-applications were applied with a 48-hr interval between respective treatments.

*Ap*, The biocontrol agent *Aureobasidium pullulans*.

*Bc*, The pathogen *Botrytis cinerea*.

*Ca*, The pathogen *Colletotrichum acutatum*.

“+” indicates application of the treatment.

“-” indicates the application of water.

*A. pullulans, B. cinerea*, and *C. acutatum* conidial suspensions were applied to all above-ground plant parts including flowers, fruits, and leaves using a hand-held sprayer. To ensure consistent application across all 1280 plants, the sprayer was calibrated for uniform output, and all conidial suspensions were prepared at standardized concentrations using a hemocytometer. The same operator applied treatments throughout the experiment under controlled greenhouse conditions. A randomized complete block design was used to minimize potential variability across blocks. Control plants were treated with a water spray only. Prior to application, Tween 20 was added to each formulation at a concentration of 0.1% to enhance coverage. Treatments were applied every seven days from the onset of flowering (week 6) until one week before the harvest period (week 12), resulting in a total of six applications over the 12-week experimental period.

### 2.4 Scoring of disease symptoms

Twelve weeks after potting the plants, ripe strawberries were harvested from each treatment group to evaluate the effect of *A. pullulans* on fruit shelf life, with consideration given to cultivar variations. The harvested fruits were placed into sales boxes and incubated at 4°C for two weeks, following the protocol established by Hokkanen et al. (2008), which has been used in numerous studies of strawberry disease development (Iqbal et al., 2022; 2023a). The effect of the various treatments on shelf life was assessed by monitoring disease progression daily over the two-week incubation period using a standardized scoring scale for grey mould and anthracnose symptoms. These daily assessments provided a quantitative time-course of symptom development for each fruit, allowing direct comparison between control and *A. pullulans*-treated fruits. A modified disease scale, as described in previous studies (Adikaram et al., 2002; Iqbal et al., 2021; Miller-Butler et al., 2019), was used for the scoring: 0 = no fungal growth, 1 = fungal growth only on the margin of any lesions, 2 = even but slight fungal growth all over the fruit, and 3 = dense fungal growth all over the fruit. While disease severity was monitored using this scale, only the time (days) until which fruits reached a score of 1 (indicating disease onset) was used for analysis of shelf life. Shelf life was defined as the number of days from harvest until a fruit reached a disease score of 1, which was considered the onset of visible disease.

### 2.5 Statistical analyses

Treatments were compared pairwise by filtering the data for two treatments and estimating a two-way ANOVA using a block model. The model included treatment (two levels in filtered data), cultivar (16 levels), the interaction between treatment and cultivar, and block as an additive factor. This analysis was repeated for selected pairs of treatments relevant to testing the concept of B-IPM. See **Table 3** for the pairs of treatments and corresponding hypotheses that were statistically compared. Effects were tested using F-tests based on type 2 sums of squares. The main reason for filtering the data and modelling treatments pairwise was to simplify the calculation and comparison of treatment responses between cultivars. These treatment responses were derived from the ANOVA model by calculating the estimated marginal means for each treatment and cultivar, then computing the difference between the treatment means for each cultivar. These mean differences were then compared using a Tukey post-hoc test, with differences considered significant where p-values were below 0.05. Model assumptions for the two-way ANOVA with block model were tested using the Shapiro-Wilk test for normality and the Breusch-Pagan test for homoscedasticity. Normality tests indicated some minor normality issues (p-values between 0.05 and 0.01) which were deemed acceptable due to the large sample size (approximately 200 observations, with some variation due to missing values). Homoscedasticity tests indicated one case of clear significance (p < 0.001). In this case, the model was verified by comparing the results to those of a model where the response variable was transformed using the square-root transformation. This transformation resulted in a non-significant test for homoscedasticity, identical significant results from F-tests, and similar results from post-hoc tests. Statistical analysis was conducted in R (ver. 4.4.0) using R Studio (ver. 2024.04.2 + 764). The following packages were utilised: tidyverse, readxl, car, emmeans, and multcomp. The raw data used for these analyses are provided as an Excel file in the **Supplementary Materials**.

**Table 3:** Overview of the pairwise treatment comparisons and associated hypotheses tested in this study.

Pairwise comparisons	Hypotheses
T1 vs. T8	Fruits from plants treated with biocontrol (T1) experience longer shelf life than fruits from untreated control plants (T8) and the effect size is genotype specific.
T2 vs. T4	Fruits from plants pre-treated with biocontrol before inoculation with the pathogen *B. cinerea* experience longer shelf life than fruits from control plants inoculated with the same pathogen, but the effect size is genotype specific.
T5 vs. T4	Fruits from plants post-treated with biocontrol after inoculation with the pathogen *B. cinerea* experience longer shelf life than fruits from control plants inoculated with the same pathogen, but the effect size is genotype specific.
T3 vs. T6	Fruits from plants pre-treated with biocontrol before inoculation with the pathogen *C. acutatum* experience longer shelf life than fruits from control plants inoculated with the same pathogen, but the effect size is genotype specific.
T7 vs. T6	Fruits from plants post-treated with biocontrol after inoculation with the pathogen *C. acutatum* experience longer shelf life than fruits from control plants inoculated with the same pathogen, but the effect size is genotype specific.
T4 vs. T6	Control plants inoculated with the pathogens *B. cinerea* or *C. acutatum* show various degrees of resistance. Further, plant genotypes showing high resistance to one pathogen also show high resistance to the second, and vice versa.

For detailed information on the treatments, see **Table 2** in Materials and Methods.

## 3 Results and discussion

We started the quest for B-IPM by screening a Swedish collection of 16 wild strawberry genotypes for resistance to the cosmopolitan pathogens *B. cinerea* and *C. acutatum*. Eight treatments including controls and pre/post sprayings with the beneficial biocontrol agent *A. pullulans*, were applied to a well-replicated setup involving 1280 individually-treated potted plants. Grey mould and anthracnose disease are postharvest diseases and, as *B. cinerea* spores are more or less omnipresent, disease symptoms typically emerge on harvested strawberries regardless of whether or not the plants were experimentally inoculated with pathogenic spores. Hence, even fruits in the overall control treatment (T8) showed emerging grey mould symptoms, although they appeared later than the disease symptoms shown by plants experimentally inoculated with *B. cinerea* (T4) or *C. acutatum* spores (T6) (**Supplementary Figures S1**-**S4**; **Supplementary Table S1**).

**Figure 1:**
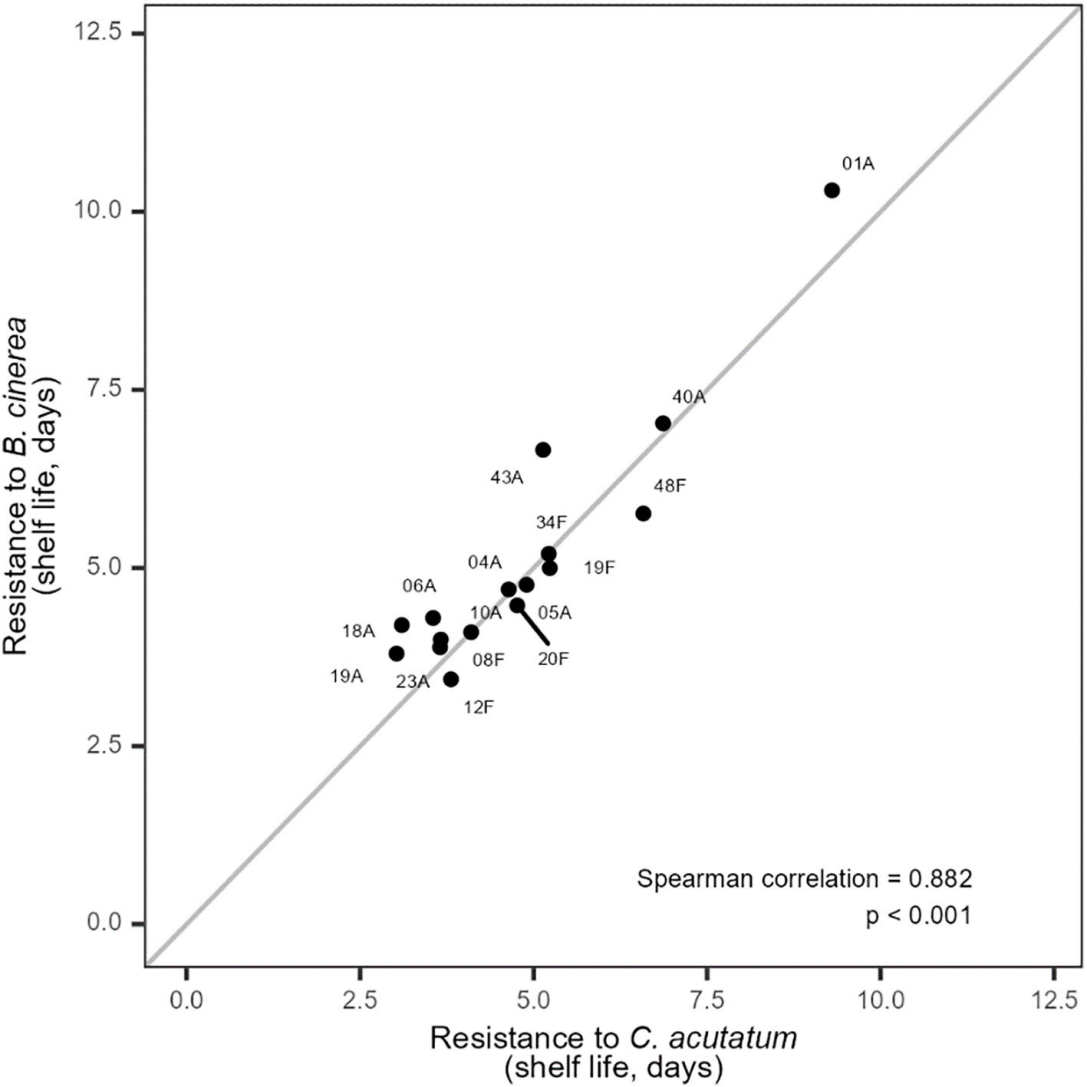
Scatterplot showing the relationship between plant resistance to *Botrytis cinerea* (T4) and *Colletotrichum acutatum* (T6) in 16 wild strawberry genotypes. Confidence intervals for these values are provided in **Supplementary Figure S3** to show variability among replicates.

As predicted (**Table 3**), control plants (without biocontrol treatments) inoculated with *B. cinerea* (T4) and *C. acutatum* (T6) showed very high variation in their intrinsic resistance to these pathogens (**Figure 1**). Some plant genotypes, including genotype 01A, showed particularly remarkable resistance, with a shelf life of approximately 10 days without biocontrol. This is the longest symptom-free period we have observed for wild strawberry inoculated with *B. cinerea* or *C. acutatum*. Resistance to the two pathogens was highly correlated across genotypes, suggesting that some wild strawberry lines may possess traits conferring resistance to multiple pathogens simultaneously (**Figure 1**; **Supplementary Table S1**). Importantly, genotype 01A, which expressed the strongest resistance to both pathogens, also demonstrated high compatibility with the biocontrol agent *A. pullulans*, providing proof-of-concept for B-IPM. Such trait expression could be influenced by factors like fruit surface chemistry, nutrient composition, or other genotype-specific attributes that affect microbial colonization (Chen et al., 2016; Droby et al., 2009). The important point here is that the absence of a clear trade-off between resistance and facilitation among genotypes suggests that these traits may be optimised independently, thus paving the way for B-IPM strategies. Such resistance traits are highly valued in plant breeding (O’Hara et al., 2024), and, as *F. vesca* is frequently used for rewilding cultivated Rosaceae crops, these resistance resources —especially promising genotypes like 01A — could contribute meaningfully to the development of more sustainable crop protection strategies in strawberry breeding. Nonetheless, this remains to be further explored through molecular or physiological studies.

**Figure 2:**
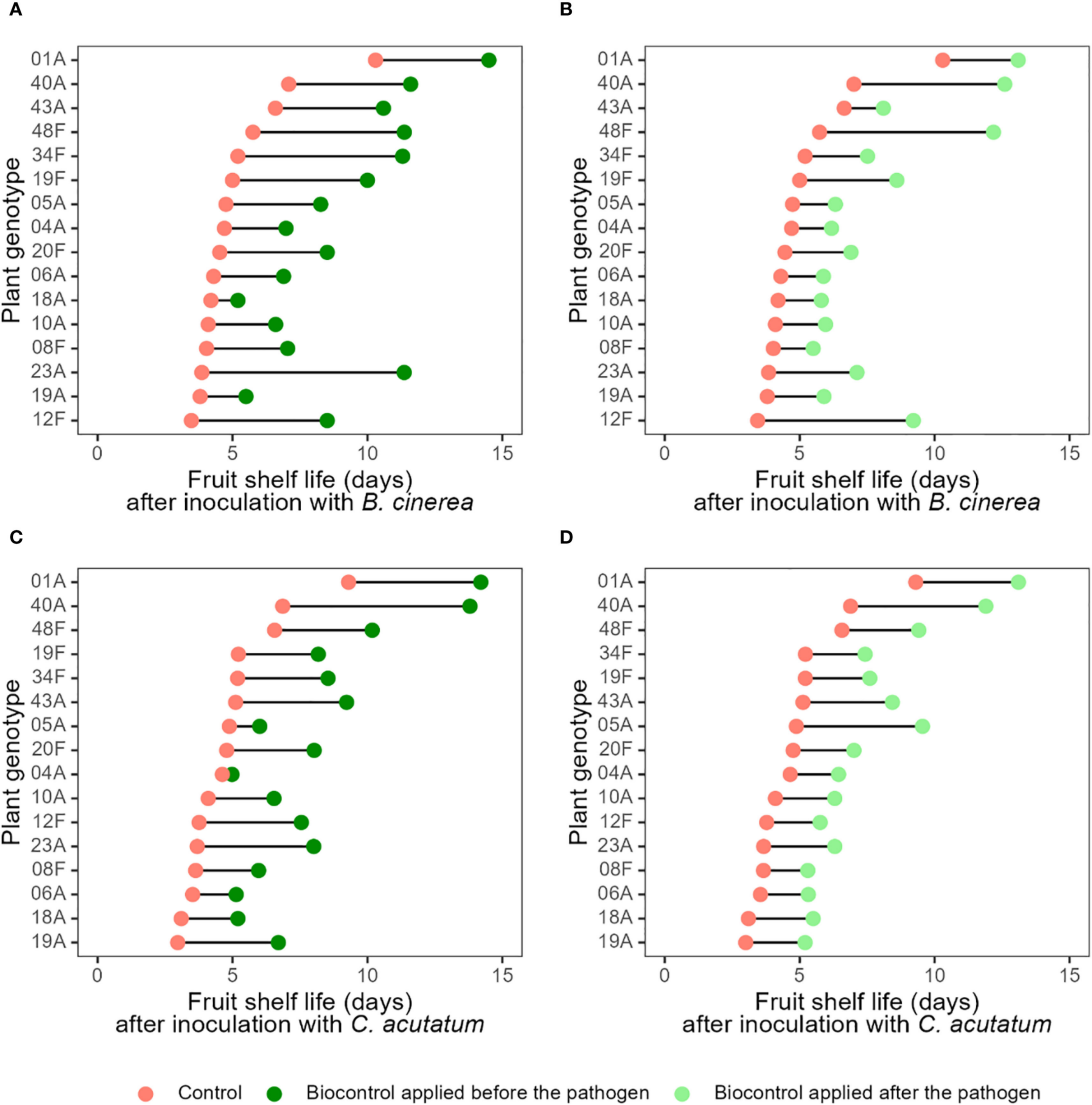
Mean fruit shelf life (number of days to develop symptoms of grey mould and anthracnose disease) for 16 strawberry genotypes without and with biocontrol treatment. The distance between paired points reflects the shelf-life extension provided by the biocontrol agent *Aureobasidium pullulans* for each plant genotype. A greater distance between points indicates stronger effect of the treatment, while a shorter distance suggests a smaller effect. Red points denote the shelf life of fruit from control plants (without biocontrol), while dark and light green points represent biocontrol treatment with *A. pullulans* applied 48 hours before and after pathogen, respectively. **(A–D)** show pairwise comparisons for treatments **(A)** T2 vs T4; **(B)** T5 vs T4, **(C)** T3 vs T6; and **(D)** T7 vs T6, respectively. Treatment descriptions are provided in **Table 2**. Variability among replicates, expressed as confidence intervals, is shown in **Supplementary Figure S3**.

The biocontrol agent (*A. pullulans* AP SLU-6) contributed to extending the shelf life of most strawberry genotypes, irrespective of whether the biocontrol treatment was applied before (**Figures 2A, C**; **Supplementary Figures S1**-**S4**) or after (**Figures 2B, D**; **Supplementary Figures S1**-**S4**) inoculation with the pathogens, although its effect was greater when applied 48 hours prior to pathogen inoculation rather than after it. These encouraging effects confirm previous studies which have shown that *A. pullulans* contribute greatly to controlling both *B. cinerea* and *C. acutatum* on cultivated strawberries in greenhouses (Iqbal et al., 2021; 2022) and in commercial field settings (Iqbal et al., 2023b). Repeated inoculations with *B. cinerea* and *C. acutatum* over the 6-week period did not visibly reduce overall plant viability, and all genotypes continued to flower and produce fruit. Minor foliar lesions and localised necrosis were occasionally observed at inoculation sites, but these symptoms did not appear to affect fruit set under our experimental conditions. Although fruit set and plant biomass were not quantitatively measured, our observations suggest that repeated pathogen inoculations over did not substantially impair plant performance.

Importantly, however, the effect size of the biocontrol treatment varied widely between plant genotypes (**Figure 2**; **Supplementary Figures S1**-**S4**). For example, when compared with their respective control treatments, *A. pullulans* application improved the shelf life of plants inoculated with *B. cinerea* by just 1 day for the least compatible genotype, 18A, but by 7.5 days for the most compatible genotype, 23A (**Figure 2**; **Supplementary Figures S1**-**S4**). Similar ranges of biocontrol efficacy were found for plant genotypes inoculated with *C. acutatum* (**Figure 2**; **Supplementary Figures S1**-**S4**). Generally, biocontrol seems to extend the disease-free period for both pathogens most effectively in plant genotypes 01A, 12F, 19F, 23A, 40A, and 48F, with a lesser effect on the other genotypes (**Figure 2**; **Supplementary Figures S1**-**S4**; **Supplementary Table S1**). Interestingly, plants’ resistance to the pathogens seems unrelated to their ability to facilitate biocontrol (**Figure 2**; **Supplementary Figures S1**-**S4**). This is encouraging, as it suggests that resistance to pathogens and the ability to facilitate biocontrol are independent traits, meaning that they could be independently optimised in breeding programmes. These results contradict the “popular truth” widely held by phytopathologists which presumes a negative relationships between resistance and biocontrol (Chen et al., 2015; Grau Nersting et al., 2006; Nerva et al., 2022; Park et al., 2023; Pérez-Jaramillo et al., 2016). In fact, the strawberry genotype with by far the strongest resistance (genotype 1A) also showed a very good ability to facilitate biocontrol, enhancing the shelf life of this already durable genotype by a further ca. 5 days. Figuratively speaking, this 5-day extension represents an eternity for strawberry growers and retailers who are constantly challenged by postharvest diseases which cause high levels of food loss over a short period. While this study was designed to provide the first proof-of-concept of B-IPM, we acknowledge that uncovering the underlying mechanisms is essential to implement it in practical breeding. The observed independence between resistance and facilitation traits suggests that these are regulated by distinct genetic pathways. Our findings provide a strong phenotypic foundation to guide such efforts and help identify elite genotypes suitable for breeding programs. In this context, B-IPM emerges as a promising strategy to align crop improvement with the broader agricultural goals of reducing chemical inputs and achieving more sustainable plant protection. Extending this concept further, it is noteworthy that *C. acutatum* is a hemibiotrophic pathogen, initiating infection through a biotrophic phase before transitioning to necrotrophy. The effects observed here may therefore partly reflect interference with its early biotrophic stage. Moreover, recent evidence demonstrates that *A. pullulans* can also suppress strictly biotrophic pathogens; for instance, Remolif et al. (2024) reported significant inhibition of *Entyloma belangeri*, the causal agent of white haze disease in apple. Together with its well-documented activity against diverse pathogens such as *Aspergillus carbonarius*, *Greeneria uvicola*, and *Rhizoctonia solani* (Alimzhanova et al., 2025; Di Francesco et al., 2021; Rathnayake et al., 2018), these findings indicate that *A. pullulans* utilise broad-spectrum mechanisms potentially effective across trophic modes. Recognizing this broader applicability reinforces the potential of the B-IPM approach and highlights the value of breeding genotypes that can facilitate biocontrol against a wide array of pathogens.

B-IPM offers a strategic breeding framework to integrate resistance and biocontrol compatibility into a unified crop improvement approach. It aims to develop plant varieties that combine durable pathogen resistance with the capacity to support beneficial microbial agents, thereby enhancing the effectiveness of integrated pest management (IPM) systems. This approach relies on the selection and integration of genotypes that express both traits —either independently or synergistically and can be further supported by molecular breeding tools once trait-linked loci are identified. B-IPM thus offers an adaptable strategy for developing next-generation cultivars tailored to sustainable, low-input agricultural systems.

## 4 Conclusions

The relevance of B-IPM is underscored by current global efforts to reduce dependence on chemical pesticides. There is not yet a single silver bullet for crop-protection that can replace chemical pesticides when these are phased out across the world (Stenberg, 2017). Therefore, many states and political unions (including the EU) prescribe that IPM, combining several preventive methods, must be applied to minimise the need for pesticides (European Commission, 2020). While plant resistance and biocontrol stand out as by far the best non-chemical methods, the lack of integration in how they have so far been used has slowed down the much-needed green transformation of agriculture (Deguine et al., 2021). The results of this study offer a solution to this problem, as they show that plant resistance and biocontrol can contribute additive effects to pathogen control and that some plant genotypes can simultaneously maximise plant resistance and facilitate biocontrol. This opens up the potential for match-making between plants and beneficial microbes and suggests that breeding for integrated pest management (B-IPM) may provide a key to the pesticide-free agriculture that has for so long seemed unrealistic.

## Data Availability

The original contributions presented in the study are included in the article/**Supplementary Material**. Further inquiries can be directed to the corresponding author.
