# Supplementary material for: Breeding for Integrated Pest Management (B-IPM): a new concept simultaneously optimising plant resistance and biocontrol
Source: Front Plant Sci. 2025 Sep 16;16:1659069. doi: 10.3389/fpls.2025.1659069 (PMC12481220; doi:10.3389/fpls.2025.1659069)
Supplement: Supplementary file 1 [file DataSheet1.docx]

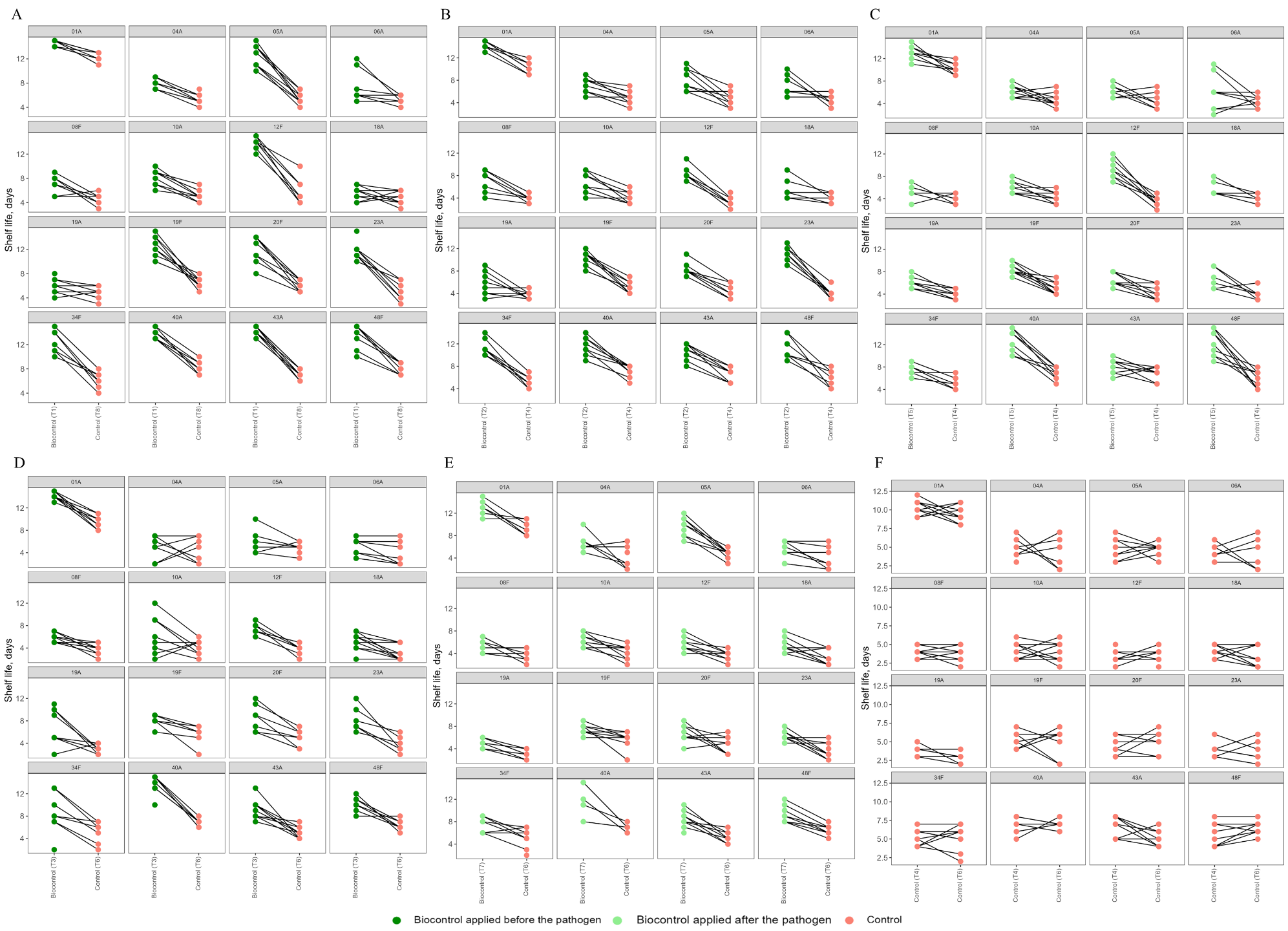


**Supplementary Fig. 1**. Fruit shelf life (number of days to develop symptoms of grey mould and anthracnose disease) for 16 strawberry genotypes without and with biocontrol treatment. Each facet in a panel represents one of the 16 genotypes, with green points on the left indicating the shelf life of fruits treated with biocontrol (Aureobasidium pullulans) and red points on the right showing the shelf life of fruits in the control treatment. Lines connect the paired observations from the same block, highlighting the within-block comparison between the two treatments. Dark green points represent A. pullulans applied 48 hours before Botrytis cinerea or Colletotrichum acutatum inoculation, while light green points indicate A. pullulans applied 48 hours after either B. cinerea or C. acutatum inoculation. Panels A-F show pairwise comparisons for treatments (A), T1 vs T8; (B), T2 vs T4, (C); T5 vs T4; (D), T3 vs T6; (E), T7 vs T6; (F), T4 vs T6 (see Table 3). Treatment descriptions are provided in Table 2.


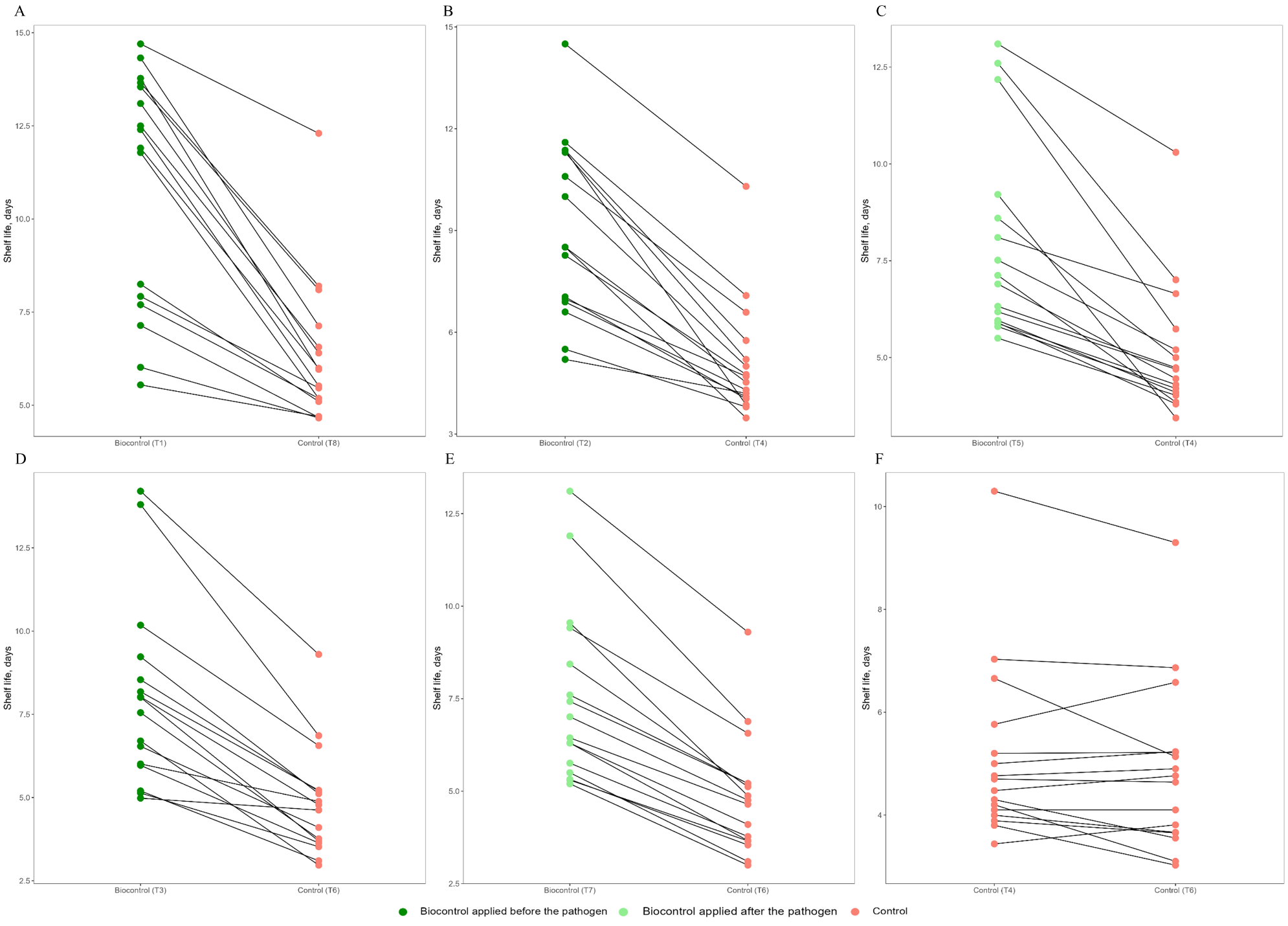


**Supplementary Fig. 2.** Mean fruit shelf life (number of days to develop symptoms of grey mould and anthracnose disease) for 16 strawberry genotypes without (red points) and with (green points) biocontrol treatment. Each panel include mean shelf-life values for each of the 16 plant genotypes, with lines connecting the mean values for the same cultivar across the two treatments. Dark green points represent biocontrol (using A. pullulans) applied 48 hours before Botrytis cinerea or Colletotrichum acutatum inoculation, while light green points indicate biocontrol applied 48 hours after either B. cinerea or C. acutatum inoculation. Panels A-F show pairwise comparisons for treatments (A), T1 vs T8; (B), T2 vs T4, (C); T5 vs T4; (D), T3 vs T6; (E), T7 vs T6; (F), T4 vs T6 (see Table 3). Treatment descriptions are provided in Table 2.


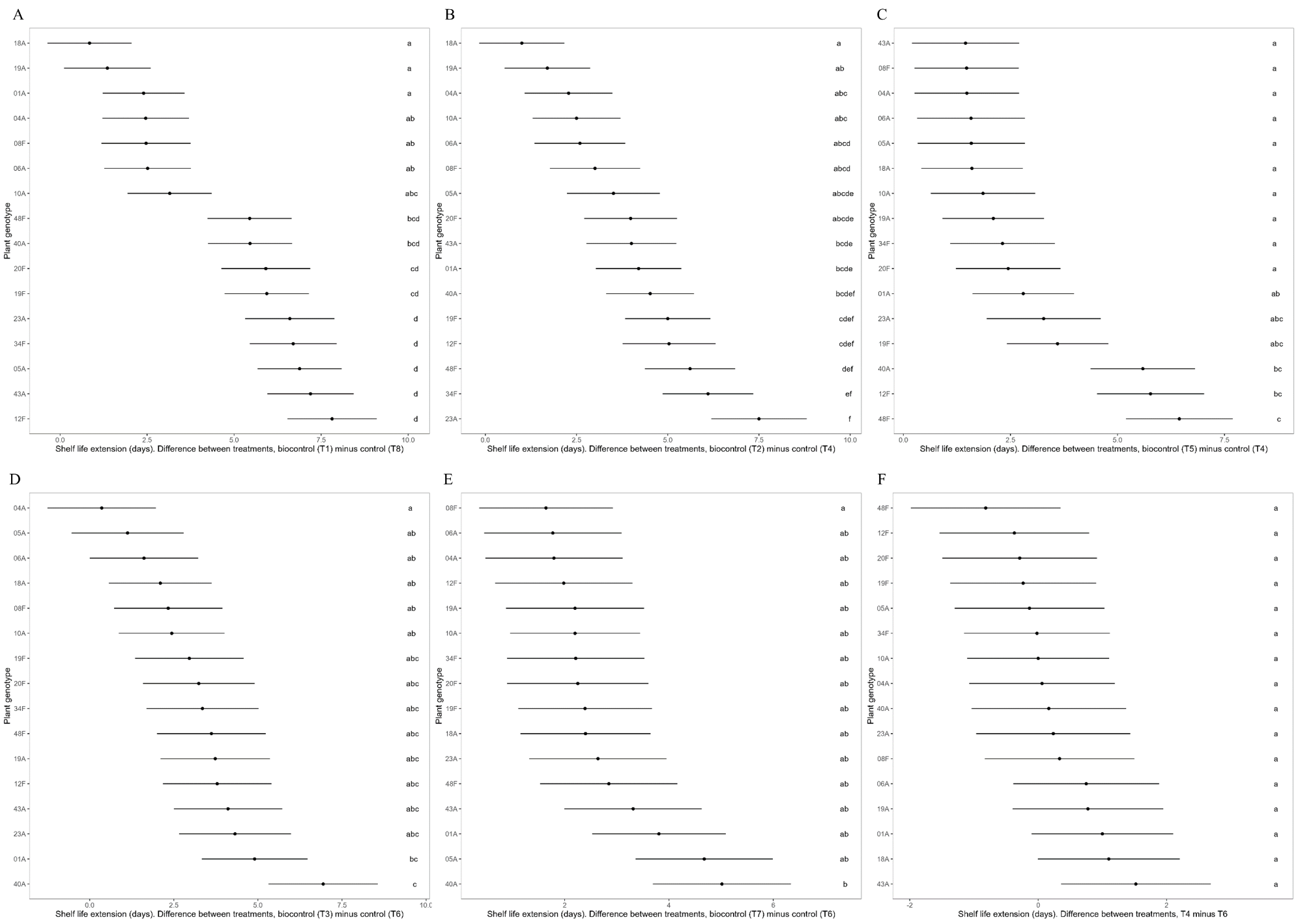


**Supplementary Fig. 3.** Confidence intervals for the difference in fruit shelf life between treatments for each of the 16 strawberry genotypes. Values close to zero suggest that the application of Aureobasidium pullulans has minimal effect (i.e., no additive effect to plant resistance) on shelf life. The significance letters to the right indicate results from pairwise comparisons of cultivars: plant genotypes sharing a letter show similar responses to A. pullulans while genotypes with no shared letters show significantly different responses. Panels A-D show pairwise comparisons for treatments (A), T1 vs T8; (B), T2 vs T4, (C); T5 vs T4; (D), T3 vs T6; (E), T7 vs T6; (F), T4 vs T6 (see Table 3). Treatment descriptions are provided in Table 2.


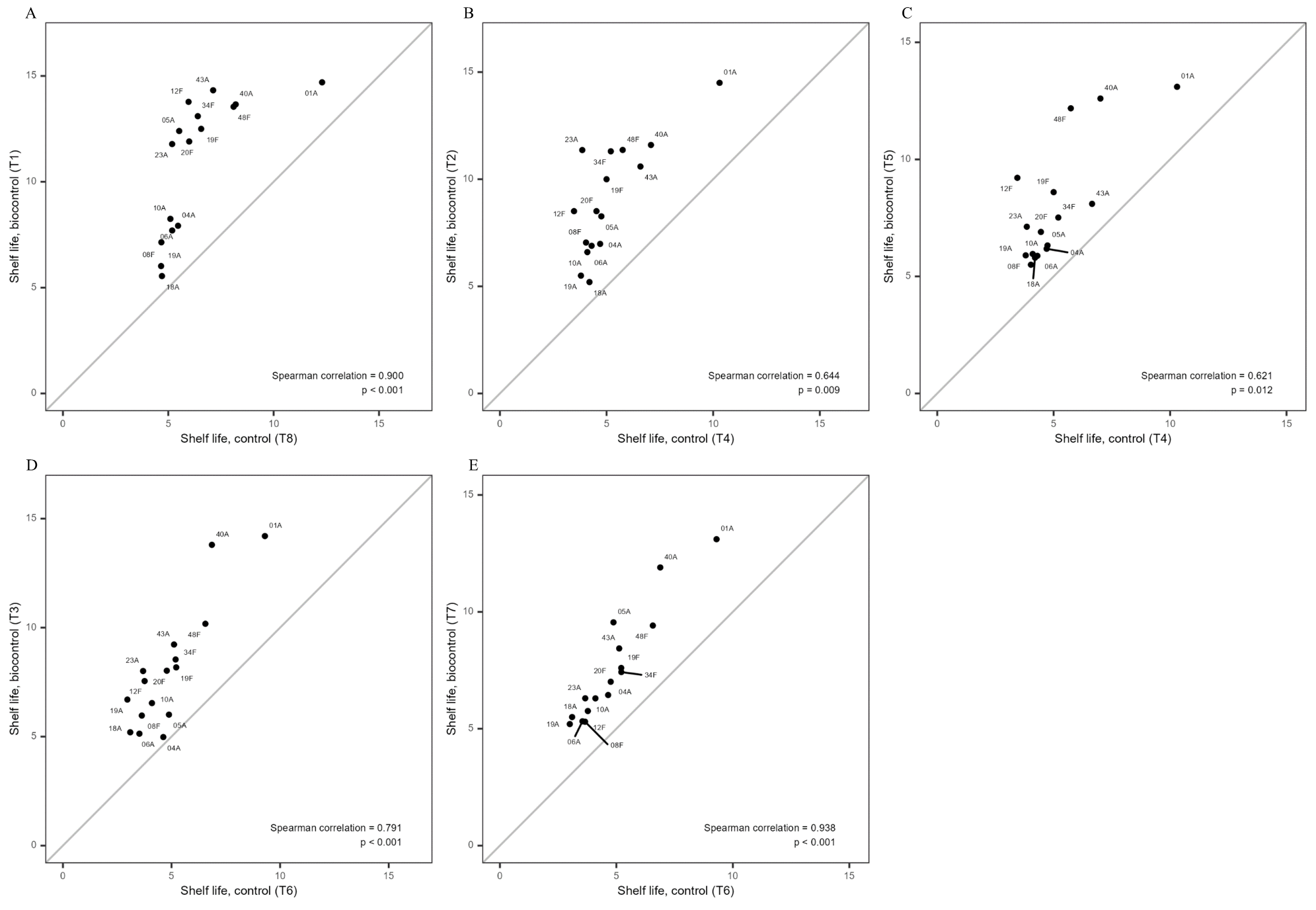


**Supplementary Fig. 4:** Scatterplots showing fruit shelf life (i.e., number of days to develop symptoms of grey mould and anthracnose disease) for 16 strawberry genotypes. The x-axes show shelf life values for the control treatments (without biocontrol), while the y-axes show shelf life values with biocontrol (with A. pullulans) treatment. The diagonal lines indicate equal values for both axes. Points above the diagonal line correspond to cases in which biocontrol enhanced shelf life, i.e., contributed an additive effect to plant resistance. Panels A-E show pairwise comparisons for treatments (A) T1 vs T8; (B) T2 vs T4; (C) T5 vs T4; (D) T3 vs T6; (E), and T7 vs T6 (see Table 3). Treatment descriptions are provided in Table 2.
