# Supplementary material for: Breeding for Integrated Pest Management (B-IPM): a new concept simultaneously optimising plant resistance and biocontrol
Source: Front Plant Sci. 2025 Sep 16;16:1659069. doi: 10.3389/fpls.2025.1659069 (PMC12481220; doi:10.3389/fpls.2025.1659069)
Supplement: Supplementary file 2 [file Table1.docx]

**Table S1**. Analysis of variance (ANOVA) of the shelf life (number of days until harvested fruit in sales boxes develop symptoms of grey mould and anthracnose disease) of strawberry genotypes exposed to different treatments in a block design.

| Combination Source of variation | | Sum sq | Df | F value | Pr(>F) |
| --- | --- | --- | --- | --- | --- |
| T1 vs T8 | Treatment | 1494.4 | 1 | 838 | 0.000 |
|  | Genotype | 1569 | 15 | 58.7 | 0.000 |
|  | Block | 9.4 | 9 | 0.6 | 0.806 |
|  | Treatment: Genotype | 364.1 | 15 | 13.6 | 0.000 |
|  | Residuals | 447.6 | 251 |  |  |
| T2 vs T4 | Treatment | 1083 | 1 | 613.7 | 0.000 |
|  | Genotype | 1195.3 | 15 | 45.2 | 0.000 |
|  | Block | 51.8 | 9 | 3.3 | 0.001 |
|  | Treatment: Genotype | 199.7 | 15 | 7.5 | 0.000 |
|  | Residuals | 446.5 | 253 |  |  |
| T5 vs T4 | Treatment | 594 | 1 | 327.7 | 0.000 |
|  | Genotype | 1179.8 | 15 | 43.4 | 0.000 |
|  | Block | 12.2 | 9 | 0.7 | 0.667 |
|  | Treatment: Genotype | 193.7 | 15 | 7.1 | 0.000 |
|  | Residuals | 469.4 | 259 |  |  |
| T3 vs T6 | Treatment | 720.9 | 1 | 239.6 | 0.000 |
|  | Genotype | 1289.6 | 15 | 28.6 | 0.000 |
|  | Block | 16.9 | 9 | 0.6 | 0.776 |
|  | Treatment: Genotype | 166.4 | 15 | 3.7 | 0.000 |
|  | Residuals | 743.1 | 247 |  |  |
| T7 vs T6 | Treatment | 528.9 | 1 | 264.2 | 0.000 |
|  | Genotype | 1111.1 | 15 | 37 | 0.000 |
|  | Block | 11.9 | 9 | 0.7 | 0.745 |
|  | Treatment: Genotype | 69.1 | 15 | 2.3 | 0.004 |
|  | Residuals | 506.4 | 253 |  |  |
| T4 vs T6 | Treatment | 5.1 | 1 | 3.3 | 0.072 |
|  | Genotype | 772.1 | 15 | 32.8 | 0.000 |
|  | Block | 6.6 | 9 | 0.5 | 0.895 |
|  | Treatment: Genotype | 26.8 | 15 | 1.1 | 0.323 |
|  | Residuals | 399 | 254 |  |  |
